# First Molecular Evidence of *Babesia vogeli, Babesia vulpes,* and *Theileria ovis* in Dogs from Kyrgyzstan

**DOI:** 10.3390/pathogens12081046

**Published:** 2023-08-15

**Authors:** Kursat Altay, Ufuk Erol, Omer Faruk Sahin, Mehmet Fatih Aydin, Ayperi Aytmirzakizi, Nazir Dumanli

**Affiliations:** 1 Department of Parasitology, Faculty of Veterinary Medicine, Sivas Cumhuriyet University, 58140 Sivas, Türkiye; ufukerol@cumhuriyet.edu.tr (U.E.); ofsahin@cumhuriyet.edu.tr (O.F.S.); 2 Department of Public Health, Faculty of Health Sciences, Karamanoğlu Mehmetbey University, 70100 Karaman, Türkiye; mfaydin@kmu.edu.tr; 3 Faculty of Veterinary Medicine, Kyrgyz-Turkish Manas University, Bishkek 720044, Kyrgyzstan; ayperi.aytmirzakyzy@gmail.com; 4 Department of Parasitology, Faculty of Veterinary Medicine, Firat University, 23119 Elazig, Türkiye; ndumanli2@yahoo.com

**Keywords:** *B. vogeli*, *B. vulpes*, PCR, DNA sequence, dog, Kyrgyzstan

## Abstract

Tick-borne parasitic diseases cause mild to severe infections among vertebrate hosts, including dogs. Species in the genus *Babesia* are important tick-borne pathogens and have worldwide distributions. Although there are data on the prevalence and distribution of *Babesia* species among dogs around the world, there is no information available in Kyrgyzstan, according to a literature review. In this study, 337 dogs were screened by nested PCR for the presence of the *18S small subunit ribosomal RNA* (*18S SSU rRNA*) gene of piroplasm species. Overall prevalence was 6.23% (21/337) for *Babesia*/*Theileria* spp. DNA sequencing of positively tested samples revealed that eighteen samples were infected with *Babesia vogeli* (*B. vogeli*) (5.34%), two samples with *B. vulpes* (0.59%), and one sample with *Theileria ovis* (*T. ovis*) (0.29%). The phylogenetic analyses and nucleotide sequences in contrast with those present in GenBank revealed that two nucleotide substitutions (594th and 627th) were found between *B. vogeli* isolates, including ours, indicating that the mutation is relatively rare. The sequences of other pathogens obtained in this study confirmed 100% nucleotide identity with *B. vulpes* and *T. ovis* sequences in GenBank. To the best of our knowledge, *B. vogeli*, *B. vulpes*, and *T. ovis* were detected for the first time in dogs from Kyrgyzstan, and it is thought that results will contribute to the understanding of the epidemiology of canine tick-borne pathogens in the country.

## 1. Introduction

Babesiosis is a tick-borne protozoan disease of various vertebrate hosts, including humans and domestic animals caused by the genus *Babesia* (Phylum Apicomplexa, Class Piroplasmea, and Order Piroplasmida). The parasite is naturally transmitted by ixodid ticks, causing a hemolytic disease by invading the host erythrocytes. The clinical symptoms of the disease depend on species, even strains, and host related factors such as age, immune status, and the presence of co-infections [1]. While the species generally have host-specific behavior, piroplasm species known to be host-specific have been detected in other hosts in recent years. However, the epidemiological and clinical significance of this condition are not clear yet. For these reasons, studies have been carried out in recent years to investigate *Babesia* species in different host species and to understand the taxonomy and epidemiology of these species. This has led to an increased interest in the *Babesia* species in the world [2,3,4].

Canine babesiosis was described as caused by *Babesia canis* in 1895 in Italy for the first time [5]. Recently, the existence of three different species under *B. canis* (*B. canis canis, B. canis vogeli,* and *B. canis rossi*) has been revealed. There are those who consider them subspecies of *B. canis* due to their morphological similarities, as well as different species due to their geographical distribution, vector diversity, genetic characteristics, and differences in the clinical symptoms they cause [6]. *Babesia vogeli* is transmitted by *Rhipicephalus sanguineus* (*R. sanguineus*), and accepted as a globally distributed pathogen. *Babesia canis* is transmitted by *Dermacentor reticulatus* (*D. reticulatus*), and the parasite is prevalent in European countries with warm climates. The parasite has also been detected in Asian countries [6,7]. *Babesia rossi*, transmitted with *Haemaphysalis lechi* (*Hae. lechi*)*,* is a species limited to Africa [8]. The clinical course of infections caused by these parasites, apart from their different vectors, also differs [9,10]. *Babesia gibsoni*, described in 1910 in India, is the second *Babesia* species introduced in dogs [11]. Canine *Babesia* species have been defined for many years according to the size of the agent in the erythrocyte. Accordingly, large parasites (3–5 µm) were named as *B. canis* and small parasites (1.5–2.5 µm) as *B. gibsoni* [10]. Although the trophozoite and meront forms of *B. gibsoni* are generally smaller than those of *B. canis*, they fall into the same cluster according to their molecular characterization [12]. *Babesia vulpes* and *B. conradae* are other small *Babesia* species infecting dogs [13]. In addition, several unclassified species of *Babesia* can infect dogs [14,15]. On the other hand, species such as the equine parasites *B. cabballi* and *T. equi* [2] and the cattle parasite *T. annulata* have been detected in dogs by molecular methods [3].

Different identification techniques, such as microscopic, serologic, and molecular, have been used for the detection of blood parasites such as *Babesia* and *Theileria* in hosts. Microscopic methods, such as blood smears stained with Giemsa stain, are much preferred by researchers because they are easy to perform and do not need expensive laboratory equipment and chemicals. However, since the diagnosis of the pathogens using this method is performed according to the morphological characteristics of the pathogens, species with similar morphological characteristics cannot be distinguished from each other. Furthermore, microscopic techniques are not eligible for the detection of chronic cases due to the low amount of pathogens in the blood samples [2,9]. Serological methods, especially immunofluorescent antibody testing, are used to diagnose piroplasmosis in hosts. However, in acute piroplasmosis infections, the low or absence of specific antibodies in the blood may cause failure to identify the disease [3]. Moreover, due to cross-reactions between *Babesia* species, serologic methods may fail to identify the species or genotype causing the disease [14]. The above-mentioned limitations of microscopic and serological methods in the diagnosis of piroplasmosis have led to the need for different methods in the diagnosis of the disease. Molecular-based methods have many advantages compared with microscopic and serologic methods, such as the detection of low amounts of pathogen DNA present in the samples, the identification of pathogen species levels, and the diagnosis of acute or chronic infections. In addition, DNA sequence analysis can detect the genetic diversity of pathogens and can also contribute to understanding the taxonomy of the piroplasm species [1,2,4,7,15].

To date, no record of canine babesiosis has been found in the literature in Kyrgyzstan. Recently, we reported the first molecular presence and prevalence of *Dirofilaria immitis* (*D. immitis*) and *D. repens* [16], canine and bovine hemotropic mycoplasma species [17,18], and canine hepatozoonozis in dogs from Kyrgyzstan [19]. On the other hand, the presence of *T. orientalis, T. annulata, B. major* [20,21], *Anaplasma centrale* (*A. centrale*)*, A. phagocytophilum*-like 1, *A. capra*, and *A. ovis* were revealed in cattle and sheep [22,23]. In this study, we aimed to detect *Babesia* species infecting shelter dogs in Kyrgyzstan and to confirm the species of the parasite detected through sequencing. In our study, we also tried to reveal the existence of other species of piroplasm that can infect dogs based on the *18S SSU rRNA* gene.

## 2. Materials and Methods

### 2.1. Study Area, Collection of Blood Samples, and DNA Extraction

**Figure 1:**
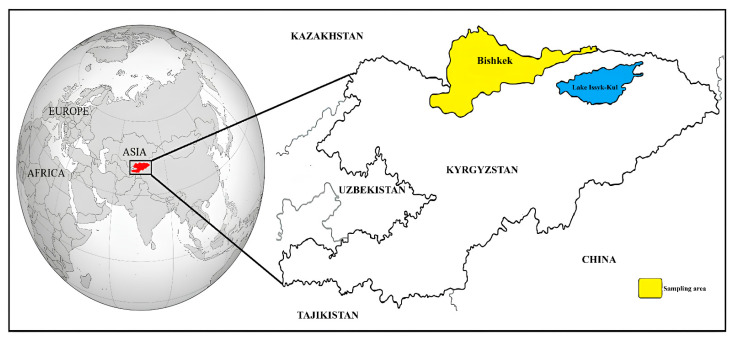
Location of Kyrgyzstan in the world and sampling area.

The Republic of Kyrgyzstan is a country in Central Asia that is bordered by Uzbekistan, Kazakhstan, Tajikistan, and China. The country has seven managerial regions: Chuy, Jalal-Abad, Talas, Naryn, Batken, Issyk-Kul, and Osh. Bishkek, located in the Chuy region, is the country’s largest city and capital (Figure 1). Bishkek is located in the north of Kyrgyzstan, bordering Kazakhstan. The country has a continental climate, with hot summers and cold winters, and is surrounded by mountains. In this study, DNA samples were obtained from a study conducted by our team [16] and these DNA samples were stored in Sivas Cumhuriyet University Parasitology Department Laboratories under appropriate conditions. To summarize, the blood samples were collected from 337 shelter dogs in Bishkek between 2017 and 2019. In the sampling process, cooperation was made with the Kyrgyz-Turkish Manas University Veterinary Teaching Hospital. All dogs showed no clinical symptoms at first observation and were recorded as healthy or asymptomatic. Total DNA isolation from the blood samples was conducted using a commercial DNA isolation kit (PureLink Genomic DNA Kit, Invitrogen, Carlsbad, CA, USA).

### 2.2. Polymerase Chain Reaction (PCR), Sequencing, and Phylogenetic Analyses

A total of 337 blood samples were analyzed by nested PCR in terms of piroplasm species. The names, sequence, amplicon sizes, and PCR conditions of primers used in this study are listed in Table 1.

PCR was carried out at 25 µL final volume, including 14.875 µL DNase-RNase-free sterile water (Qiagen^®^, Hilden, Germany), 2.5 µL 10× PCR buffer (Thermo Scientific™, Vilnus, Lithuania), 2.5 µL MgCl_2_ (25 mM) (Thermo Scientific™, Vilnus, Lithuania), 0.5 µL dNTP (10 mM) (Cat.No.: R0181, Thermo Scientific™, Vilnus, Lithuania), 0.125 µL of Taq DNA polymerase (5 U) (Cat.No.: EP0402, Thermo Scientific™, Vilnus, Lithuania), 1 µL (10 pmol/µL) of each of the primers, and 2.5 µL template DNA. For nested PCR, 1 µL of the first round of PCR product was used as template DNA. After PCR amplifications, the PCR product was loaded on the agarose gel and subjected to electrophoresis at 90 V for 60 min. The agarose gel was stained with an ethidium bromide solution for 20 min and checked for ~500 bp amplicons using a UV transilluminator.

In this study, all PCR positive samples were sequenced with BJ1 and BN2 primers. DNA sequences were carried out with an ABI 3730XL analyzer (Applied Biosystems, Foster City, CA, USA) using the BigDye Terminator v3.1 Cycle Sequencing Kit (Applied Biosystems, Foster City, CA, USA). Nucleotide sequence files were opened with FinchTV (version 1.4.0) software (Geospiza Inc., Seattle, Washington, DC, USA) for the determination of chromatogram quality. Sequences with low chromatogram quality were not used for the determination of the consensus sequences. MEGA-11 software (Version 11.0.13) was used to determine and assemble the consensus sequences [26]. The *B. vogeli, B. vulpes,* and *T. ovis* consensuses identified in this study were aligned with reference sequences of the mentioned pathogens present in the GenBank using the MUSCLE algorithm in the MEGA-11 software [26]. Further nucleotide identities between our sequence and sequences present in GenBank were determined by BLAST analyses.

After the determination of the consensus sequence of *B. vogeli, B. vulpes, and T. ovis* isolates identified in the current study, these sequences were uploaded to GenBank under the following accession numbers: *B. vogeli* OR116199-OR116216, *B. vulpes* OR116236-OR116237, and *T. ovis* OR116238. The lengths of consensus sequence recorded in the GenBank for *B. vogeli, B. vulpes, and T. ovis* were 450–455 bp, 481 bp, and 461 bp, respectively.

Phylogenetic trees were constructed using maximum likelihood analysis (ML) in the MEGA-11 software [26] for the determination of genetic relationships between pathogens identified in this study and those present in the GenBank. Before constructing the trees, the best-fit model for ML was determined as the Kimura-2 + G parameter model for *Theileria* species [27] using the Find Best-Fit Substitution Model in MEGA-11 [26]. Bootstrap values were performed with 1000 replicates.

## 3. Results

**Figure 2:**
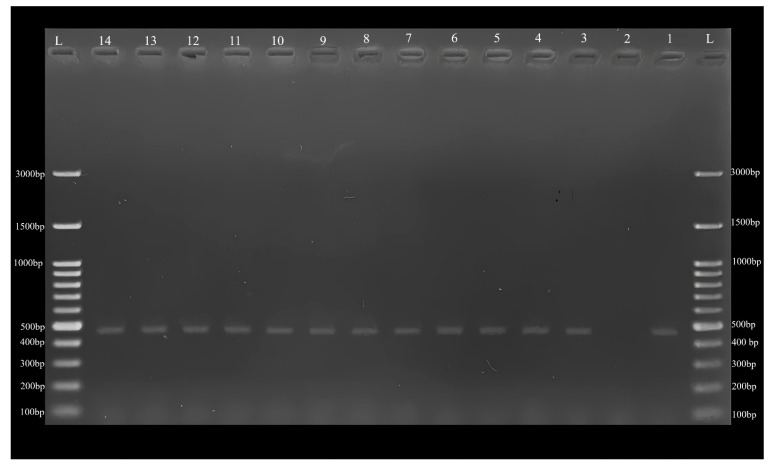
Agarose-gel electrophoresis of positive samples obtained in this study. L. Ladder, 1. Positive control, 2. Negative control, 3–14. Positive samples.

As a result of PCR analysis of 337 blood samples, 21 (6.23%) samples were found to be positive (Figure 2) for piroplasm species. All PCR positive samples were sequenced for species identifications of pathogens detected in this study.

In this study, 18 samples (5.34%) were found to be infected with *B. vogeli* using DNA sequence analyses. The partial sequence of the *18S SSU rRNA* gene of *B. vogeli* isolates identified in this study showed 100% nucleotide identity among each other. Furthermore, BLAST analyses revealed that 98.03–100% nucleotide identities were present between our *B. vogeli* isolates and other *B. vogeli* isolates uploaded to GenBank from different parts of the world. *Babesia vogeli* isolates identified in this work shared 100% nucleotide identity with *B. vogeli* isolates detected from dogs in Egypt (AY371197, OP604258, and OP604259) and dogs in Romania (HQ662635 and JF461252). Phylogenetic analyses of the *18S SSU rRNA* gene revealed that single nucleotide polymorphisms (SNPs) were present in two sites between *B. vogeli* isolates. The 594th and 627th nucleotides are cytosine in both our *B. vogeli* isolates and those from Egypt (AY371197, OP604258, and OP604259) and Romania (HQ662635 and JF461252). These nucleotides are thymine in *B. vogeli* isolates obtained from dogs in China (MK881091), Brazil (MW62732), Spain (AY150061), Paraguay (MH100719), and Taiwan (HQ148664), from ticks in China (OK663019) and Malawi (OQ727064), from red fox in France (MK674799), and from cat in China (MN067709).

The DNA of *B. vulpes* was detected in 2 out of 337 dog samples (0.59%) in this study. The 98.97–100% nucleotide identities were found between *B. vulpes* isolates obtained in this work and *B. vulpes* detected in red foxes, dogs, and tick species present in GenBank. *B. vulpes 18S SSU rRNA* gene sequences shared 100% nucleotide identity with each other and *B. vulpes* isolates identified from red foxes in Italy (MK742780), Poland (MH553357), Türkiye (MF040153), Slovakia (KY175167), and China (MW192450), and from dogs in Hungary (MH544242, MW805763), and from *R. sanguineus* in Portugal (MN207196).

Unexpectedly, *T. ovis* DNA was detected in one (0.29%) dog in the current study, according to DNA sequence analysis. The BLAST analysis showed that 100% nucleotide identity was present between *T. ovis* dog isolates identified in the current work and *T. ovis* isolates identified from *R. evertsi evertsi* (OQ766977) in Ghana, cattle (LC714842) in Iraq, goat (OM666861) in India, *Ornithodoros lahorensis* (*O. lahorensis*) (OM181705) and Tibetian sheep (MZ047355) in China, *Hyalomma marginatum* (*Hy. marginatum*) (OM066225) in Türkiye, and sheep (MZ604123) in India deposited to the GenBank from various parts of the world.

**Figure 3:**
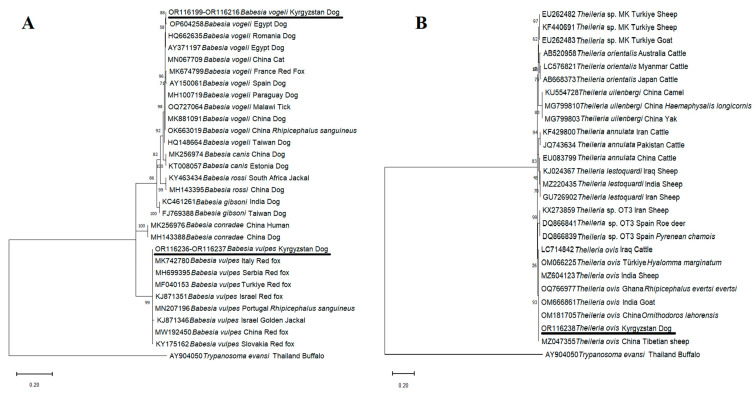
Phylogenetic trees according to *18 SSU rRNA* sequences of *Babesia* (**A**) and *Theileria* (**B**) species. The figure was created using the ML method. Bootstrap values were performed with 1000 replicates. The evolutionary history was performed using the ML method and the Kimura-2 + G model was used with *Babesia* and *Theileria* species, respectively [27]. Evolutionary analyses were conducted in MEGA-11 [26]. *Babesia* and *Theileria* species identified in this study are underlined in the phylogenetic tree. *Trypanosoma evansi* was added as outgroup. Underlined sequences were identified in this study.

Phylogenetic trees revealed that our *B. vogeli*, *B. vulpes*, and *T. ovis* isolates were grouped with *B. vogeli, B. vulpes,* and *T. ovis* isolates, respectively, and placed in a different cluster with other *Babesia* (Figure 3A) and *Theileria* species (Figure 3B).

## 4. Discussion

Babesiosis is one of the most important tick-borne infectious diseases of dogs. Canine babesiosis is an infection that causes a wide range of symptoms in dogs, ranging from subclinical to severe. In most cases in dogs, the non-specific clinical manifestations include fever, lethargy, loss of appetite, jaundice, pale mucous membranes due to acute hemolysis, splenomegaly, and weight loss. In addition to these clinical symptoms, the diseases can cause death in cases of severe infection. The severity of the disease mainly depends on the pathogenicity of the species or subspecies [28,29]. Both the clinical signs are not fully descriptive in the diagnostic attempts of veterinarians, and the difficulties in identifying the species found in this lineage have been overcome by molecular methods. PCR and sequencing are widely used in diagnosis and in determining the phylogenetic positions of species [22,23,30]. Studies using these methods have provided more accurate information about the epidemiology of the disease as well as the identification of species. Disease agents, which were known in the past as two types as large and small species (*B. canis* and *B. gibsoni*), are now understood to include at least six species (*B. canis canis, B. canis vogeli, B. canis rossi, B. gibsoni, B. vulpes,* and *B. condradae*) [5,6,11,13]. In fact, some species of other vertebrates have been identified in dogs with these methods [4]. On the other hand, it is very important to know about the presence and prevalence of pathogens in order to know what diseases can emerge in each country. In this study, the investigation of piroplasm parasites in dogs from Kyrgyzstan was performed by PCR and sequence analysis for the first time. In PCR, we amplified the *18S SSU rRNA* gene of *Babesia* species. PCR positive results were seen in 21 (6.23%) of 337 blood samples. As a result of the sequencing, we have revealed the presence of *B. vogeli* and *B. vulpes* in dogs in the country. Interestingly, the sequence results revealed the presence of *T. ovis* in dogs. It is a sheep protozoon parasite transmitted by ticks [31]. The results showed that babesiosis should be taken into account in dog health in the country, as well as contributing to the global epidemiology of the parasites. In addition, it may be thought that dogs may contribute to the epidemiology of *T. ovis* [4].

*Babesia vogeli* is a globally distributed tick-borne pathogen, and this species has been detected on five continents. This species can be referred to as the most common species of *Babesia* [14,32,33,34,35,36]. In this study, *B. vogeli* was detected in 18 (5.34%) of 337 blood samples. The prevalence of the parasite was 2.1% in Malaysia [37], 4.8% in Brazil [38], 5.1% in Egypt [39], 5.1% in Iraq [40], 8.5% in Bosnia and Herzegovina [41], 9.3% in Iran [42], and 10.8% in Nigeria [43]. When the studies given above were evaluated, it was seen that the prevalence of *B. vogeli* was quite different. It is possible to associate this with many factors, such as the characteristics of the geographical region, the size of the sampling, whether the animals studied are pets, shelter animals or stray animals, and the method used. The important aspect of these studies is that the parasite is usually first detected in the region. In our study, *B. vogeli* was introduced for the first time to dogs in Kyrgyzstan. It is thought that these results will contribute to the global epidemiology of the parasite. In this study, all *B. vogeli* isolates were sequenced, and the 98.03–100% nucleotide identities were determined between our *B. vogeli* isolates and other isolates identified from dogs, red foxes, cats, and various tick species. According to the nucleotide sequence comparison of the partial *18S SSU rRNA* gene region of between *B. vogeli* isolates, SNPs were found in two sites. The 594th and 627th nucleotides are cytosine in both our *B. vogeli* isolates and *B. vogeli* isolates identified in Egypt (AY371197, OP604258, and OP604259) and Romania (HQ662635 and JF461252). These nucleotides are thymine in *B. vogeli* isolates in China (MK881091), Brazil (MW62732), Spain (AY150061), Paraguay (MH100719), Taiwan (HQ148664), China (OK663019), Malawi (OQ727064), France (MK674799), and China (MN067709) (Figure 3A). Studies revealed that the *18S SSU rRNA* gene was highly conserved, with little intraspecific variation [44]. Therefore, two nucleotide substitutions in small parts of the *18S SSU rRNA* gene of *B. vogeli* isolates, when evaluated together with the data in the GenBank, suggested that the pathogen may consist of two different genotypes.

Banet et al. [1] showed that *T. annae, B. annae, B. microti*-like piroplasm, *B.* cf. *microti*, and *Babesia* Spanish dog isolates are synonymous with *B. vulpes*. For this reason, the species detected in this study was named as *B. vulpes* [1]. Although it is stated that *B. vulpes* is mainly transmitted by *Ixodes* species, this issue has not been fully elucidated. Since *I. hexoganus* is more common in parasite-infected dogs, it is suggested that its vector may be this species [45]. In addition, parasite DNA has also been detected in tick species such as *I. ricinus* [46], *D. reticulatus* [47], and *R. sanguineus* [48]. *Ixodes crenulatus* has been identified as the dominant tick species in rodents [49]. The presence of *D. marginatus. D. niveus, R. sanguineus, R. turanicus, Hae. punctata, Hae. sulcata, Hy. marginatum, Hy. anatolicum, Hy. scupense,* and *Hy. asiaticum* have also been recorded in the country [50]. *Babesia vulpes* infects foxes and dogs [51]. However, there are many studies on the prevalence of *B. vulpes* in red foxes almost all over the world, and in these studies, the prevalence of *B. vulpes* in foxes has been detected at 72.00% in Spain [52], 69.20% in Portugal [53], 50.70% in Austria [54], 46.40% in Germany [55], and 30.70% in Poland [56]. While there is a high fox population in Kyrgyzstan [57], further studies on foxes may also find the parasite widespread. However, it is known that the parasite does not cause clinical infection in foxes, but it causes clinical disease in dogs [58]. There have been limited studies on the prevalence of *B. vulpes* in dogs. Studies are generally in the form of case reports. In this way, it has been reported in countries such as Canada [59], Spain [60], and Russia [61]. In North America, 9098 dogs were examined and *B. vulpes* was detected in only 0.52% of them [58]. In Spain, 153 dogs were investigated and *T. annae* (*B. vulpes*) was detected in 0.70% of the samples [62]. In this study, we detected *B. vulpes* in 0.59% of the 337 dogs examined. This prevalence, together with other data, shows that the prevalence of *B. vulpes* in dogs is quite low. As a result, as the detection of the parasite in the country will guide future studies, it has emerged that *B. vulpes* should be considered in clinical cases associated with babesiosis in dogs. The high nucleotide identities (98.97–100%) were seen between our *B. vulpes* isolates and *B. vulpes* identified from different hosts, such as red foxes, dogs, and tick species. This result shows that the *18S SSU rRNA* gene can be used successfully in the diagnosis of *B. vulpes* among hosts, but the gene is not useful for the determination of the genetic diversity of the pathogen.

The improvement of molecular identification techniques and the widespread use of these for the detection of parasitic diseases, especially in the last two decades, have provided important contributions to research into the high specificity and sensitivity diagnosis of parasitic diseases, the determination of the genetic diversity of pathogens, and the detection of new species/strains/genotypes [15,22,23,30]. Furthermore, molecular techniques can provide unexpected species detection in different host species [63]. Normally, the BJ1 and BN2 primers used in this study were thought to be specific to *Babesia* species [25], but subsequent studies have shown that these primers also amplify *Theileria* species [64]. Therefore, all PCR-positive samples were sequenced with the primers, and *T. ovis* was detected in one dog sample in the current study. The *T. ovis* dog isolate identified in this study was recorded to the GenBank (Accession number: OR116238). This isolate was included among Genbank registered *T. ovis* isolates in the phylogenetic tree, as confirmed by sequence analysis (OR116238). The 100% nucleotide identity was detected between the *T. ovis* dog isolate detected in this study and *T. ovis* isolates identified from different hosts, including sheep and tick species detected in different parts of the world. No study has been found in Kyrgyzstan that has been conducted among sheep and goats, which are the hosts of this parasite, to which we compared our obtained sequence for the determination of genetic diversity between *T. ovis* isolates. Beforehand, *T. ovis, T. buffeli* (*T. orientalis* complex), and *T. luwenshuni* were detected by molecular methods in asymptomatic dogs in Iran [4]. The study in Iran was carried out on shepherd dogs, and they detected *T. luwenshuni, T. buffeli,* and *T. ovis* in 13% of the 52 dogs they examined [4]. This result is considerably higher than our result. In this case, the most decisive factor may be shepherd dogs. Additionally, *T. annae* (*B. microti*-like), *Theileria* sp. OT3, *T. equi*, and *T. annulata* have been detected in dogs [2,65,66,67]. Although these findings do not prove that dogs are hosts for the specified species, they may be interpreted as contributing to the epidemiology of these species. This is even more important considering that dogs are used in the management of herds, especially in species such as *T. ovis*, which is a parasite of sheep and goats.

## 5. Conclusions

To the best of our knowledge, this study presents the first molecular detection of *B. vogeli, B. vulpes*, and *T. ovis* in dogs from Kyrgyzstan. These results may contribute to better understanding the epidemiology of canine babesiosis in Kyrgyzstan. Moreover, the *B. vogeli* nucleotide sequence revealed that this species may consist of two genotypes (according to Genbank data), and we think this data might be clarified with further studies. However, there is a need to carry out studies involving wild Canidae and vector tick species in the country.

## Figures and Tables

**Table 1:** Information about the primers used in this study.

	Primer Name	Sequence (5′-3′)	Amplicon Size	PCR Conditions	References
Outer PCR	Nbab_1F	AAGCCATGCATGTCTAAGTATAAGCTTTT	1600 bp	−94 °C 5 min. −94ºC 1 min., 56 °C 1 min., and 72 °C 1 min. (35 cycle) −72 °C 7 min.	[24]
	Nbab_1R	CTTCTCCTTCCTTTAAGTGATAAGGTTCAC			
Inner PCR	BJ1	GTCTTGTAATTGGAATGATGG	~500 bp	−94 °C 5 min. −94 °C 1 min., 56 °C 1 min., and 72 °C 1 min. (35 cycle) −72 °C 7 min.	[25]
	BN2	TAGTTTATGGTTAGGACTACG			

## Data Availability

Data available in a publicly accessible repository.

## References

[1] Baneth G., Florin-Christensen M., Cardoso L., Schnittger L. (2015). Reclassification of *Theileria annae* as *Babesia vulpes* sp. nov. Parasite. Vector..

[2] Beck R., Vojta L., Mrljak V., Marinculić A., Beck A., Živičnjak T., Cacciò S.M. (2009). Diversity of *Babesia* and *Theileria* species in symptomatic and asymptomatic dogs in Croatia. Int. J. Parasitol..

[3] Bigdeli M., Rafie S.M., Namavari M.M., Jamshidi S. (2012). Report of *Theileria annulata* and *Babesia canis* infections in dogs. Comp. Clin. Path..

[4] Gholami S., Laktarashi B., Shiadeh M.M., Spotin A. (2015). Genetic variability, phylogenetic evaluation and first global report of *Theileria luwenshuni*, *T. buffeli*, and *T. ovis* in sheepdogs in Iran. Parasitol. Res..

[5] Piana G.P., Galli-Valerio B. (1895). Su di un’ infezione del cane con parasiti endoglobulari. Mod. Zooiatr..

[6] Matijatko V., Torti M., Schetters T.P. (2012). Canine babesiosis in Europe: How many diseases?. Trends Parasitol..

[7] Aktas M., Ozubek S. (2017). A survey of canine haemoprotozoan parasites from Turkey, including molecular evidence of an unnamed *Babesia*. Comp. Immunol. Microbiol. Infect. Dis..

[8] Uilenberg G. (2006). Babesia—A historical overview. Vet. Parasitol..

[9] Uilenberg G., Franssen F.F.J., Perie N.M., Spanjer A.A.M. (1989). Three groups of *Babesia canis* distinguished and a proposal for nomenclature. Vet. Quart..

[10] Solano-Gallego L., Baneth G. (2011). Babesiosis in dogs and cats-expanding parasitological and clinical spectra. Vet. Parasitol..

[11] Patton W.S. (1910). Preliminary report on a new piroplasm (*Piroplasma gibsoni* sp. nov.) found in the blood of the hounds of the Madras Hunt and subsequently discovered in the blood of the jackal *Canis aureus*. Bull. Soc. Pathol. Exot..

[12] Schnittger L., Rodriguez A.E., Florin-Christensen M., Morrison D.A. (2012). *Babesia*: A world emerging. Infect. Genet. Evol..

[13] Penzhorn B.L., Oosthuizen M.C. (2020). *Babesia* species of domestic cats: Molecular characterization has opened pandora’s box. Front. Vet. Sci..

[14] Birkenheuer A.J., Levy M.G., Breitschwerdt E.B. (2003). Development and evaluation of a seminested PCR for detection and differentiation of *Babesia gibsoni* (Asian genotype) and *B. canis* DNA in canine blood samples. J. Clin. Microbiol..

[15] Ozubek S., Aktas M. (2017). Molecular evidence of a new *Babesia* sp. in goats. Vet. Parasitol..

[16] Aydın M.F., Altay K., Aytmirzakizi A., Dumanlı N. (2020). First molecular detection of *Dirofilaria immitis* and *D. repens* in dogs from Kyrgyzstan. Acta Parasitol..

[17] Altay K., Aydın M.F., Aytmirzakizi A., Jumakanova Z., Cunusova A., Dumanlı N. (2020). First molecular evidence for *Mycoplasma haemocanis* and *Candidatus* Mycoplasma haematoparvum in asymptomatic shelter dogs in Kyrgyzstan. Kafkas Univ. Vet. Fak. Derg..

[18] Altay K., Aydın M.F., Aytmirzakizi A., Jumakanova Z., Cunusova A., Dumanlı N. (2023). First molecular detection and phylogenetic analysis of *Mycoplasma wenyonii* and *Candidatus* Mycoplasma haemobos in cattle in different parts of Kyrgyzstan. Biologia.

[19] Altay K., Aydın M.F., Aytmirzakizi A., Jumakanova Z., Cunusova A., Dumanlı N. (2019). Molecular survey of hepatozoonosis in natural infected dogs: First detection and molecular characterisation of *Hepatozoon canis* in Kyrgyzstan. Kafkas Univ. Vet. Fak. Derg..

[20] Aktaş M., Kısadere İ., Özübek S., Cihan H., Salıkov R., Cirak V.Y. (2019). First molecular survey of piroplasm species in cattle from Kyrgyzstan. Parasitol. Res..

[21] Ozubek S., Ulucesme M.C., Cirak V.Y., Aktas M. (2022). Detection of *Theileria orientalis* genotypes from cattle in Kyrgyzstan. Pathogens.

[22] Altay K., Erol U., Sahin O.F., Aytmirzakizi A. (2022). First molecular detection of *Anaplasma* species in cattle from Kyrgyzstan; molecular identification of human pathogenic novel genotype *Anaplasma capra* and *Anaplasma phagocytophilum* related strain. Ticks Tick Borne Dis..

[23] Altay K., Erol U., Sahin O.F., Aytmirzakizi A., Temizel E.M., Aydin M.F., Dumanli N., Aktas M. (2022). The detection and phylogenetic analysis of *Anaplasma phagocytophilum*-like 1, *A. ovis* and *A. capra* in sheep: *A. capra* divides into two genogroups. Vet. Res. Commun..

[24] Oosthuizen M.C., Zweygarth E., Collins N.E., Troskie M., Penzhorn B.L. (2008). Identification of a novel *Babesia* sp. from a sable antelope (*Hippotragus niger* Harris, 1838). J. Clin. Microbiol..

[25] Casati S., Sager H., Gern L., Piffaretti J.C. (2006). Presence of potentially pathogenic *Babesia* sp. for human in *Ixodes ricinus* in Switzerland. Ann. Agric. Environ. Med..

[26] Tamura K., Stecher G., Kumar S. (2021). MEGA11: Molecular evolutionary genetics analysis version 11. Mol. Biol. Evol..

[27] Kimura M. (1980). A simple method for estimating evolutionary rate of base substitutions through comparative studies of nucleotide sequences. J. Mol. Evol..

[28] Schetters T.P., Kleuskens J.A.G.M., Scholtes N., Gorenflot A. (1998). Parasite localization and dissemination in the *Babesia*-infected host. Ann. Trop. Med. Parasitol..

[29] Birkenheuer A.J., Greene C.E. (2012). Babesiosis. Green’s Infectious Diseases of the Dog and Cat.

[30] Sahin O.F., Erol U., Altay K. (2022). Buffaloes as new hosts for *Anaplasma capra*: Molecular prevalence and phylogeny based on *gtlA*, *groEL*, and *16S rRNA* genes. Res. Vet. Sci..

[31] Aktas M., Altay K., Dumanli N. (2006). PCR-based detection of *Theileria ovis* in *Rhipicephalus bursa* adult ticks. Vet. Parasitol..

[32] Criado A., Martinez J., Buling A., Barba J.C., Merino S., Jefferies R., Irwin P.J. (2006). New data on epizootiology and genetics of piroplasms based on sequences of small ribosomal subunit and cytochrome b genes. Vet. Parasitol..

[33] Criado-Fornelio A., Martinez-Marcos A., Buling-Sarana A., Barba-Carretero J.C. (2003). Molecular studies on *Babesia*, *Theileria* and *Hepatozoon* in southern Europe. Part II: Phylogenetic analysis and evolutionary history. Vet. Parasitol..

[34] Matjila P.T., Penzhorn B.L., Bekker C.P.J., Nijhof A.M., Jongejan F. (2004). Confirmation of occurrence of *Babesia canis vogeli* in domestic dogs in South Africa. Vet. Parasitol..

[35] Inokuma H., Yoshizaki Y., Shimada Y., Sakata Y., Okuda M., Onishi T. (2003). Epidemiological survey of *Babesia* species in Japan performed with specimens from ticks collected from dogs and detection of new *Babesia* DNA closely related to *Babesia odocoilei* and *Babesia divergens* DNA. J. Clin. Microbiol..

[36] Jefferies R., Ryan U.M., Muhlnickel C.J., Irwin P.J. (2003). Two species of canine *Babesia* in Australia: Detection and characterization by PCR. J. Parasitol..

[37] Prakash B.K., Low V.L., Vinnie-Siow W.Y., Tan T.K., Lim Y.A.L., Morvarid A.R., AbuBakar S., Sofian-Azirun M. (2018). Detection of *Babesia* spp. in dogs and their ticks from Peninsular Malaysia: Emphasis on *Babesia gibsoni* and *Babesia vogeli* infections in *Rhipicephalus sanguineus* sensu lato (Acari: Ixodidae). J. Med. Entomol..

[38] de Oliveira Carieli E.P., da Silva V.C.L., de Lima E.R., Dias M.B.D.M.C., Fukahori F.L.P., de Azevedo Rêgo M.S., Júnior J.W.P., de Cássia Peixoto Kim P., Leitão R.S.C.S., Mota R.A. (2016). Parasitological and molecular detection of *Babesia canis vogeli* in dogs of Recife, Pernambuco and evaluation of risk factors associated. Semina. Ciências. Agrárias..

[39] Selim A., Megahed A., Ben Said M., Alanazi A.D., Sayed-Ahmed M.Z. (2022). Molecular survey and phylogenetic analysis of *Babesia vogeli* in dogs. Sci. Rep..

[40] Badawi N.M., Yousif A.A. (2020). *Babesia canis* spp. in dogs in Baghdad province, Iraq: First molecular identifcation and clinical and epidemiological study. Vet. World..

[41] Ćoralić A., Gabrielli S., Zahirović A., Stojanović N.M., Milardi G.L., Jažić A., Zuko A., Čamo D., Otašević S. (2018). First molecular detection of *Babesia canis* in dogs from Bosnia and Herzegovina. Ticks Tick Borne Dis..

[42] Khanmohammadi M., Zolfaghari-Emameh R., Arshadi M., Razmjou E., Karimi P. (2021). Molecular identifcation and genotyping of *Babesia canis* in dogs from Meshkin Shahr County, Northwestern Iran. J. Arthropod Borne Dis..

[43] Obeta S.S., Ibrahim B., Lawal I.A., Natala J.A., Ogo N.I., Balogun E.O. (2020). Prevalence of canine babesiosis and their risk factors among asymptomatic dogs in the federal capital territory, Abuja, Nigeria. Parasite Epidemiol. Control..

[44] Mandal M., Banerjee P.S., Garg R., Ram H., Kundu K., Kumar S., Kumar G.R. (2014). Genetic characterization and phylogenetic relationships based on *18S rRNA* and *ITS1* region of small form of canine *Babesia* spp. from India. Infect. Genet. Evol..

[45] Camacho A.T., Pallas E., Gestal J.J., Guitián F.J., Olmeda A.S., Telford III S.R., Spielman A. (2003). *Ixodes hexagonus* is the main candidate as vector of *Theileria annae* in Northwest Spain. Vet. Parasitol..

[46] Lledó L., Giménez-Pardo C., Dominguez-Penafiel G., Sousa R., Gegúndez M.I., Casado N., Criado A. (2010). Molecular detection of hemoprotozoa and *Rickettsia* species in arthropods collected from wild animals in the Burgos Province, Spain. Vector Borne Zoonotic Dis..

[47] Hodžić A., Zörer J., Duscher G.G. (2017). *Dermacentor reticulatus*, a putative vector of *Babesia* cf. microti (syn. Theileria annae) piroplasm. Parasitol. Res..

[48] Iori A., Gabrielli S., Calderini P., Moretti A., Pietrobelli M., Tampieri M.P., Galuppi R., Cancrini G. (2010). Tick reservoirs for piroplasms in central and northern Italy. Vet. Parasitol..

[49] Akyshova B., Chen Y.N., Chen J. (2022). Abundance of ectoparasitic ticks and mites (Acari: Ixodida, Mesostigmata, Trombidiformes) on rodents in the Alamedin Gorge of Kyrgyz Range, Kyrgyzstan. Syst. Appl. Acarol..

[50] Aknazarov B., Jetigenov E., Atabekova N., Suerkulov U., Abdumanap N. (2023). Spread of arthropod-borne infections in Kyrgyzstan. E3S Web Conf..

[51] Solano-Gallego L., Sainz Á., Roura X., Estrada-Peña A., Miró G. (2016). A review of canine babesiosis: The European perspective. Parasit. Vectors..

[52] Checa R., López-Beceiro A.M., Montoya A., Barrera J.P., Ortega N., Gálvez R., Marino V., González J., Olmeda Á.S., Fidalgo L.E. (2018). *Babesia microti*-like piroplasm (syn. Babesia vulpes) infection in red foxes (Vulpes vulpes) in NW Spain (Galicia) and its relationship with Ixodes hexagonus. Vet. Parasitol..

[53] Cardoso L., Cortes H.C.E., Reis A., Rodrigues P., Simões M., Lopes A.P., Vila-Viçosa M.J., Talmi-Frank D., Eyal O., Solano-Gallego L. (2013). Prevalence of *Babesia microti*-like infection in red foxes (*Vulpes vulpes*) from Portugal. Vet. Parasitol..

[54] Hodžić A., Mrowietz N., Cezanne R., Bruckschwaiger P., Punz S., Habler V.E., Tomsik V., Lazar J., Duscher G.G., Glawischnig W. (2018). Occurrence and diversity of arthropod-transmitted pathogens in red foxes (*Vulpes vulpes*) in western Austria, and possible vertical (transplacental) transmission of *Hepatozoon canis*. Parasitology.

[55] Najm N.A., Meyer-Kayser E., Hoffmann L., Herb I., Fensterer V., Pfister K., Silaghi C. (2014). A molecular survey of *Babesia* spp. and *Theileria* spp. in red foxes (*Vulpes vulpes*) and their ticks from Thuringia, Germany. Ticks Tick Borne Dis..

[56] Mierzejewska E.J., Dwużnik D., Koczwarska J., Stańczak Ł., Opalińska P., Krokowska-Paluszak M., Wierzbicka A., Górecki G., Bajer A. (2021). The red fox (*Vulpes vulpes*), a possible reservoir of *Babesia vulpes*, *B. canis* and *Hepatozoon canis* and its association with the tick *Dermacentor reticulatus* occurrence. Ticks Tick Borne Dis..

[57] McCarthy J.L., McCarthy K.P., Fuller T.K., McCarthy T.M. (2010). Assessing variation in wildlife biodiversity in the Tien Shan Mountains of Kyrgyzstan using ancillary camera-trap photos. Mt. Res. Develop..

[58] Barash N.R., Thomas B., Birkenheuer A.J., Breitschwerdt E.B., Lemler E., Qurollo B.A. (2019). Prevalence of *Babesia* spp. and clinical characteristics of *Babesia vulpes* infections in North American dogs. J. Vet. Intern. Med..

[59] Arsenault A.C., Foley P.M., Clancey N.P. (2022). *Babesia vulpes* in a dog from Prince Edward Island, Canada. Can. Vet. J..

[60] Unterköfler M.S., Pantchev N., Bergfeld C., Wülfing K., Globokar M., Reinecke A., Fuehrer H.P., Leschnik M. (2023). Case report of a fatal *Babesia vulpes* infection in a splenectomised dog. Parasitologia.

[61] Radyuk E., Karan L. (2020). A case of *Babesia vulpes* infection in a dog in Russia. Vet. Parasitol. Reg. Stud..

[62] Tabar M.D., Francino O., Altet L., Sánchez A., Ferrer L., Roura X. (2009). PCR survey of vectorborne pathogens in dogs living in and around Barcelona, an area endemic for leishmaniosis. Vet. Record..

[63] Ozubek S., Aktas M. (2017). Molecular and parasitological survey of ovine piroplasmosis, including the first report of *Theileria annulata* (Apicomplexa: Theileridae) in sheep and goats from Turkey. J. Med. Entomol..

[64] Laus F., Spaterna A., Faillace V., Veronesi F., Ravagnan S., Beribé F., Cerquetella M., Meligrana M., Tesei B. (2015). Clinical investigation on *Theileria equi* and *Babesia caballi* infections in Italian donkeys. BMC Vet. Res..

[65] Matjila P.T., Leisewitz A.L., Oosthuizen M.C., Jongejan F., Penzhorn B.L. (2008). Detection of a *Theileria* species in dogs in South Africa. Vet. Parasitol..

[66] Fritz D. (2010). A PCR study of piroplasms in 166 dogs and 111 horses in France (March 2006 to March 2008). Parasitol. Res..

[67] Simões P.B., Cardoso L., Araújo M., Yisaschar-Mekuzas Y., Baneth G. (2011). Babesiosis due to the canine *Babesia microti*-like small piroplasm in dogs-first report from Portugal and possible vertical transmission. Parasit. Vectors..

